# An Efficient Chosen-Plaintext Attack on an Image Fusion Encryption Algorithm Based on DNA Operation and Hyperchaos

**DOI:** 10.3390/e23070804

**Published:** 2021-06-24

**Authors:** Shuqin Zhu, Congxu Zhu

**Affiliations:** 1 School of Computer Science, Liaocheng University, Liaocheng 252059, China; shuqinzhu2008@163.com; 2 School of Computer Science and Engineering, Central South University, Changsha 410083, China

**Keywords:** security analysis, DNA coding, hyper-chaotic system, chosen-plaintext attack

## Abstract

This paper proposes a more efficient attack method on an image fusion encryption algorithm based on DNA operation and hyperchaos. Although several references have reported some methods to crack the image encryption algorithm, they are not the most efficient. The proposed chosen-plaintext attack method can break the encryption scheme with (4×*N*/*M*+1) or (*M*/(4×*N*)+1) chosen-plaintext images, which is much less than the number of chosen-plaintext images used in the previous cracking algorithms, where *M* and *N* represent the height and width of the target ciphertext image, respectively. The effectiveness of the proposed chosen-plaintext attack is supported by theoretical analysis, and verified by experimental results.

## 1. Introduction

With the rapid development of computer and communication technology, multimedia information, such as image, audio, and video, has become the main carrier of network information because of its clarity and vividness, and has been widely used and spread. Images always involve sensitive events, such as business, military, medical, and political affairs. Ensuring the security of images transmitted and stored on public networks has attracted unprecedented attention in the field of cryptography and information security. Image data has the characteristics of large amounts of data and a high correlation between adjacent pixels, so traditional encryption algorithms, such as DES and AES, are not suitable for image encryption [1,2]. The development of the chaos theory opens up a new way of encrypting images. Chaos is a kind of complex and seemingly random physical phenomenon produced by a certain nonlinear system. The sequence generated by chaos is pseudo-random, aperiodic, highly sensitive to control parameters and initial conditions, and can be generated quickly and accurately. These characteristics make the chaotic system especially suitable for image encryption. In recent years, many digital image encryption algorithms have been proposed [3,4,5,6,7,8]. In addition, some algorithms combined with other mathematical models on the basis of chaos, such as Kumar et al. [9], proposed the idea of combining deoxyribose nuclear acid (DNA) encoding with elliptic curve public key cryptosystem. Ahmad et al. [10] proposed a compression sensing and noise-tolerant image encryption scheme based on a chaotic map and orthogonal matrix. In order to improve the encryption speed, Aleksandra V et al. [11] constructed adaptive chaotic maps and proposed an image encryption algorithm based on these chaotic maps, which has a fast encryption speed.

Compared with information encryption, the main task of cryptanalysis is to study how to decipher the cryptosystem without knowing the key. It requires the cryptanalyzer to decode the secret key or the equivalent key by analyzing the security vulnerabilities of the ciphertext and encryption system without knowing the secret key, and then recover the plaintext information. According to the Kerckhoff principle, the security of the encryption system does not depend on the secrecy of the encryption system itself, but on the secret key, except for the secret key, whereby all other details about the cryptographic system should be disclosed. The work of cryptanalyzers constantly promotes cryptographers to propose new cryptographic algorithms. Therefore, these two technologies are interdependent. It is very important to cryptanalyze image encryption algorithms from the perspective of modern cryptography. Many image encryption algorithms based on chaos have been broken [12,13,14,15,16,17]. Zhou et al. [12] analyzed a novel image encryption scheme based on a modified Henon map using hybrid chaotic shift transform. In this algorithm, firstly, the plaintext image was scrambled, and then boundary pixels substitution and shift rows transformation were operated. Finally, two rounds of diffusion operation were carried out. However, the equivalent key stream of the algorithm is independent of the plaintext to be encrypted, so the equivalent key can be cracked by a chosen-plaintext attack. Zhu et al. [13] performed the cryptanalysis of a color image encryption scheme using an RT-enhanced chaotic tent map and obtained the equivalent keys of the cryptosystem by chosen-plaintext attacks. Li et al. [14] analyzed an image encryption scheme based on hybrid hyper-chaotic system and cellular automata. In order to encrypt different images with different keys, the algorithm takes the sum of the pixels of the image as part of the initial value of the chaotic map. However, Li et al. found the weakness of the algorithm and solved the equivalent keys through chosen-plaintext attacks. In 2016, an image cryptosystem based on circular inter-intra pixels bit-level permutation was proposed [15], but Zhang Yong [16] cracked the image encryption algorithm by using only a pair of chosen plain-cipher images or a pair of known plain-cipher images. Generally speaking, the main reason why the above algorithms are cracked is that the same equivalent key is used to encrypt different images, which is independent of plaintext. In order to encrypt different images with different key streams and gain the effect of “one secret at a time”, some algorithms [8,17,18] associated the hash values of images, such as message digest algorithm 5 (MD5) message digest and secure hash algorithm-256 (SHA-256) information digest with the initial value of chaos.

In [19], an image encryption algorithm based on DNA operation and hyperchaos was proposed, in which chaotic sequences generated by Chen’s hyper-chaotic system was adopted to scramble the locations of elements of the DNA coded image, and a DNA sequence addition operation was utilized to encrypt the encoded image. Later, it was found that the encryption algorithm could not resist a chosen-plaintext attack. Next, several attack algorithms to crack the image encryption algorithm were put forward. In [20], Zhang et al. proposed a chosen-plaintext attack algorithm. For an image with a size of *M*×*N* pixels, Zhang’s attack algorithm [20] makes use of (4*M*×*N*/3+1) chosen-plaintext images. In [21], Zhang et al. put forward another chosen-plaintext attack algorithm which needs (*M*+4*N*+1) chosen-plaintext images to crack the encryption algorithm. In [22], Xu et al. proposed a chosen-plaintext attack algorithm to crack the algorithm [19] by using max(*M*/3, 4*N*/3)+1 chosen-plaintext images. Nevertheless, these attack algorithms proposed in the above works need to use more chosen-plaintext images, so the attack efficiency is not high. In this paper, we propose a more efficient chosen-plaintext attack algorithm. Our algorithm only requires two chosen-plaintext images under the condition of *M* = 4*N*, and, at most, it needs (⌈4×*N*/*M*⌉+1) (if 4*N* > *M*) or (⌈*M*/(4×*N*)⌉+1) (if 4*N* < *M*) chosen-plaintext images. Here, ⌈*x*⌉ rounds the elements of *x* to the nearest integers towards infinity.

This paper is organized as follows. In Section 2, we redescribe the original encryption algorithm succinctly. In Section 3, the security defects of the original encryption algorithm are analyzed and the efficient chosen-plaintext attack algorithm is proposed. In Section 4, we demonstrate the effectiveness of chosen-plaintext attack with some experimental results. In Section 5, the conclusion of this paper is provided.

## 2. Description of the Original Encryption Algorithm

### 2.1. Chen Hyper-Chaotic System

The chaotic system used in the original algorithm is the Chen’s hyper-chaotic system, which is defined as follows:

(1) $$\{\begin{array}{c} \frac{dx}{dt}=a\left(y-x\right)\\ \frac{dy}{dt}=-xz+dx+cy-w\\ \frac{dz}{dt}=xy-bz\\ \frac{dw}{dt}=x+g \end{array}$$

**Figure 1:**
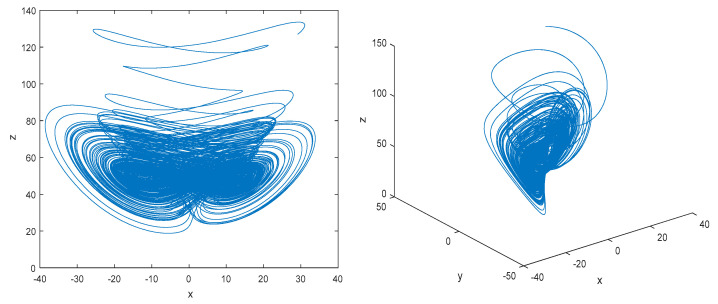
Hyper-chaotic attractors of Chen’s hyper-chaotic system with *g* = 0.54.

In Equation (1), *a*, *b*, *c*, *d*, *g* are the system parameters; when *a* =36, *b* = 3, *c* = 28, *d* = 16, and −0.7 ≤ *g* ≤ 0.7, the Chen’s hyper-chaotic system is in a hyper-chaotic state and can generate four chaotic sequences. When set *g* = 0.54 and the step size *t* = 0.001, we take the four-order Runge–Kutta method to solve the equations and obtain sequences *x*, *y*, *z*, and *w*. The hyper-chaotic attractors are show in Figure 1.

### 2.2. DNA Sequence and XOR Operation

The DNA sequence consists of four kinds of deoxynucleotides: adenine (A), thymine (T), cytosine (C), and guanine (G), in which A is paired with T, and C is paired with G. Similarly, in binary, 0 and 1 are complementary, so for two binary numbers, 00 and 11, 01 and 10 are complementary. Four bases are used: A, C, G, and T to encode 00, 01, 10, and 11, respectively. There are 24 types of encoding rules using the four bases A, C, G, and T to encode 00, 01, 10, and 11, respectively. Nevertheless, only eight of them can be seen in Table 1 which satisfy the Watson–Crick complementary rule [1]. Note that a DNA decoding rule is the reverse operation of a DNA encoding rule. For a gray image with 256 gray levels, each pixel can be encoded as a DNA sequence with the length of 4 and the encoding operation can be implemented by defining a function DNAcode (value, rule). For example, the value 184 of a pixel can be encoded as a DNA sequence “TCTG” by using the encoding rule 3, namely, DNAcode (184, 3) which outputs the result of “TCTG”. Conversely, a DNA sequence with the length of 4 can be decoded as an integer in the range of [0, 255], and the decoding operation can be expressed as a function of DNAdecode (strDNA, rule). For example, DNAdecode (“TCTG”, 3) outputs the result of 184. The original algorithm adopts encoding rules 3 and 4.

XOR operation for DNA sequences is performed according to traditional XOR in the binary. Corresponding to eight kinds of DNA encoding schemes, eight kinds of DNA XOR rules also exist, but the XOR operation rules used in the original algorithm are those shown in Table 2, which can be implemented by defining a function DNAxor (strDNA1, strDNA2). For example, DNAxor (“A”, “A”) outputs “G” and DNAxor (“C”, “T”) outputs “A”.

### 2.3. The Concrete Description of the Original Algorithm

**Figure 2:**
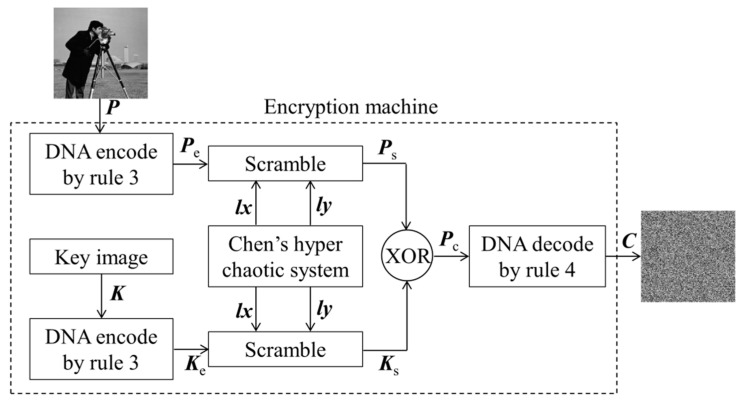
The flow chart of the original cryptosystem.

The flow of the encryption algorithm can be redescribed in Figure 2. The secret key set of the original algorithm includes the initial values (*x*_0_, *y*_0_, *z*_0_, *w*_0_) of Chen’s hyper-chaotic system and a randomly generated key image *K*. In Figure 2, *P* represents the plaintext image, and *C* represents its corresponding ciphertext image. *P*, *K* and *C* are all matrices of size *M*×*N*.

The encryption algorithm can be briefly re-described as follows:

**Step 1**: Convert image *P* and *K* into binary matrices, then carry out DNA encoding operation with rule 3 for these two binary matrices. According to Table 1, two encoded matrices *P_e_* and *K_e_* can be obtained.

**Step 2**: Generate two chaotic sequences $x={\left\{x_{i}\right\}}_{i=1}^{m}$, $y={\left\{y_{i}\right\}}_{i=1}^{4n}$ through Chen’s hyper-chaotic system under the initial condition of {*x*_0_, *y*_0_, *z*_0_, *q*_0_} and system parameters of {*a*, *b*, *c*, *d*, *k*}. Then, the two real number sequences are arranged in ascending order to obtain a row position index sequence $lx={\left\{lx\left(i\right)\right\}}_{i=1}^{m}$ and a column position index sequence $ly={\left\{ly\left(i\right)\right\}}_{i=1}^{4n}$, respectively.

**Step 3**: According to the sequences *lx* and *ly*, *P_e_* and *K_e_* are scrambled to obtain *P_s_* and *K_s_*, respectively.

(2) $$P_{s}(i,j)=P_{e}(lx(i),ly(j))$$

(3) $$K_{s}(i,j)=K_{e}(lx(i),ly(j))$$

where, *i* = 1, 2, 3, …, *M*, *j* = 1, 2, 3, …, 4*N.*

**Step 4**: DNA XOR operation is performed on *P_s_* and *K_s_* to obtain *P_c_*, according to the XOR operation rules listed in Table 2.

(4) $$P_{c}(i,j)=DNAxor(P_{s}(i,j),K_{s}(i,j))$$

**Step 5**: The fourth encoding rule which is complementary to the third encoding rule is used to decode *P_c_*, and the final ciphertext image *C* is obtained.

## 3. Security Analysis of the Original Algorithm and Chosen-Plaintext Attack

The definition of chosen-plaintext attack is as follows. In addition to not knowing the secret keys used by the cryptosystem, the attacker understands the working mechanism of the encryption algorithm and has the opportunity to use the encryption machine of the cryptosystem. Therefore, the attacker can choose some special plaintext images and obtain the corresponding ciphertext images, thereby deciphering the equivalent secret keys of the cryptosystem or the target ciphertext image.

In the whole process of encryption algorithm, the initial secret keys of the cryptosystem are not related to the image content, so the key matrices *lx*, *ly*, and *K_s_* are not changed when the image to be encrypted is varied. Therefore, the key matrices *lx*, *ly*, and *K_s_* can be cracked by chosen-plaintext attack. The key matrices *lx*, *ly*, and *K_s_* are the equivalent keys of the cryptosystem, and the virtual frame of Figure 2 is equivalent to the encryption machine of the cryptosystem.

To crack a target cipher image *CI*, our chosen-plaintext attack algorithm is divided into three stages, which will be described from Section 3.1, Section 3.2 and Section 3.3.

### 3.1. Extracting Key Matrix Ks

In order to decode the key image *K_s_*, we only need to choose one special plaintext image. The steps of the algorithm to extract key matrix *K_s_* are as follows:

**Step1**: Select a special plaintext image *P* whose pixel values are all zeros, and then use rule 3 in the Table 1 to encode *P* and to obtain the matrix *P_e_*. It is easy to know that all elements in *P_e_* are “G”.

**Step 2**: Find the ciphertext image *C* corresponding to *P* by using the encryption machine. Then, use rule 4 to encode *C* to obtain *P*_c_.

**Step 3**: Find *K_s_*. Because all elements in *P_e_* are all “G”, scrambling has no effect on *P_e_*. That is, *P_s_* = *P_e_*. According to the XOR operation rules in Table 2 and the Formula (4), the attacker can obtain the arranged key image *K_s_*. The calculation process is as follows:

(5) $$K_{s}(i,j)=\mathrm{DNAxor}(P_{s}(i,j),P_{c}(i,j))=\mathrm{DNAxor}(P_{e}(i,j),P_{c}(i,j))=P_{c}(i,j)$$

where, *i* = 1, 2, 3, …, *M*, *j* = 1, 2, 3, …, 4*N.*

### 3.2. Extracting lx and ly

From the permutation Formula (2), we can find that the essence of permutation is to exchange the rows and columns of the matrix *P*_e_. After permutation, the elements of the same row are still in the same row, and the elements of the same column are still in the same column. Taking a plain image *P_e_* of size 4×4 as an example, suppose *P_e_* and *P_s_* have the forms as

$$P_{e}=\left[\begin{array}{cccc} A & G & G & G\\ A & A & G & G\\ A & A & A & G\\ A & A & A & A \end{array}\right],\;P_{s}=\left[\begin{array}{cccc} A & A & G & A\\ A & A & G & G\\ A & A & A & A\\ G & A & G & G \end{array}\right]$$

Because the number of elements “*A*” in each row (column) of *P_e_* is different, the sequence *lx* and *ly* can be obtained by comparing the number of elements “*A*” in each row and column of *P*_s_. According to Formula (4), the elements in row *i* of *P_s_* correspond to the elements in row *lx*(*i*) of *P_e_*. Therefore, the number of “A” in row *i* of *P_s_* is equal to the number of “A” in row *lx*(*i*) of *P_e_*, *i* = 1, 2, 3, 4. As the result, it can be inferred that *lx* = [3, 2, 4, 1]. Similarly, the elements in column *j* of *P*_s_ correspond to the elements in column *ly*(*j*) of *P*_e_. Therefore, the number of “A” in column *j* of *P*_s_ is equal to the number of “A” in column *ly*(*j*) of *P*_e_, *j* = 1, 2, 3, 4. As the consequence, it can be inferred that *ly* = [2, 1, 4, 3].

Suppose the target cipher image *C* has *M* rows and *N* columns, namely, the corresponding DNA encoded image has the size of *M*×4*N*. If *M* = 4*N = L*, then the encoded image *P_e_* has *L* rows and *L* columns, which is the simplest case. In this simplest case, *lx* and *ly* can be cracked completely only through one chosen-plaintext image whose encoded image *P_e_* has the form as

(6) $$P_{e}={\left[\begin{array}{cccccc} A & G & G & G & \ldots & G\\ A & A & G & G & \ldots & G\\ A & A & A & G & \ldots & G\\ \ldots & \ldots & \ldots & \ldots & \ldots & \ldots \\ A & A & A & A & \ldots & A \end{array}\right]}_{L\times L}$$

However, if *M* < 4*N* or *M* > 4*N*, we need more chosen-plaintext images to crack all the elements in *lx* and *ly.* Let *L* = min(*M*, 4*N*), here min(*x*, *y*) returns the smaller one of *x* and *y*. The specific chosen-plaintext attack method to crack *lx* and *ly* can be described in detail as follows:

**Step 1**: Initialize *lx* as a row vector of *M* characters and *ly* as a row vector of 4*N* characters. Let *L* = min(*M*, 4*N*). The function min(*a*, *b*) returns the smallest one of *a* and *b*.

**Step 2:** Choose a special plaintext image *P* whose encoded image is *P*_e_, and a sub-image, consisting of the first *L* rows and the first *L* columns of image *P*_e_, which has the form of Equation (6). Namely, row 1 has only one character of “A” at the first column, row 2 has two characters of “A” at the first two columns, …, row *L* has *L* characters of “A” at the first *L* columns, and all of the remaining elements are “G”. By acquiring its corresponding cipher image *C*, one can obtain its corresponding image *P_c_*. Then, find the image *P_s_* by using *P_c_* and the known (cracked) image *K_s_*. By comparing *P_s_* and *P_e_*, we can obtain *L* elements of *lx* and *L* elements of *ly*. If *M* = 4*N = L*, then the attack algorithm is over.

**Figure 3:**
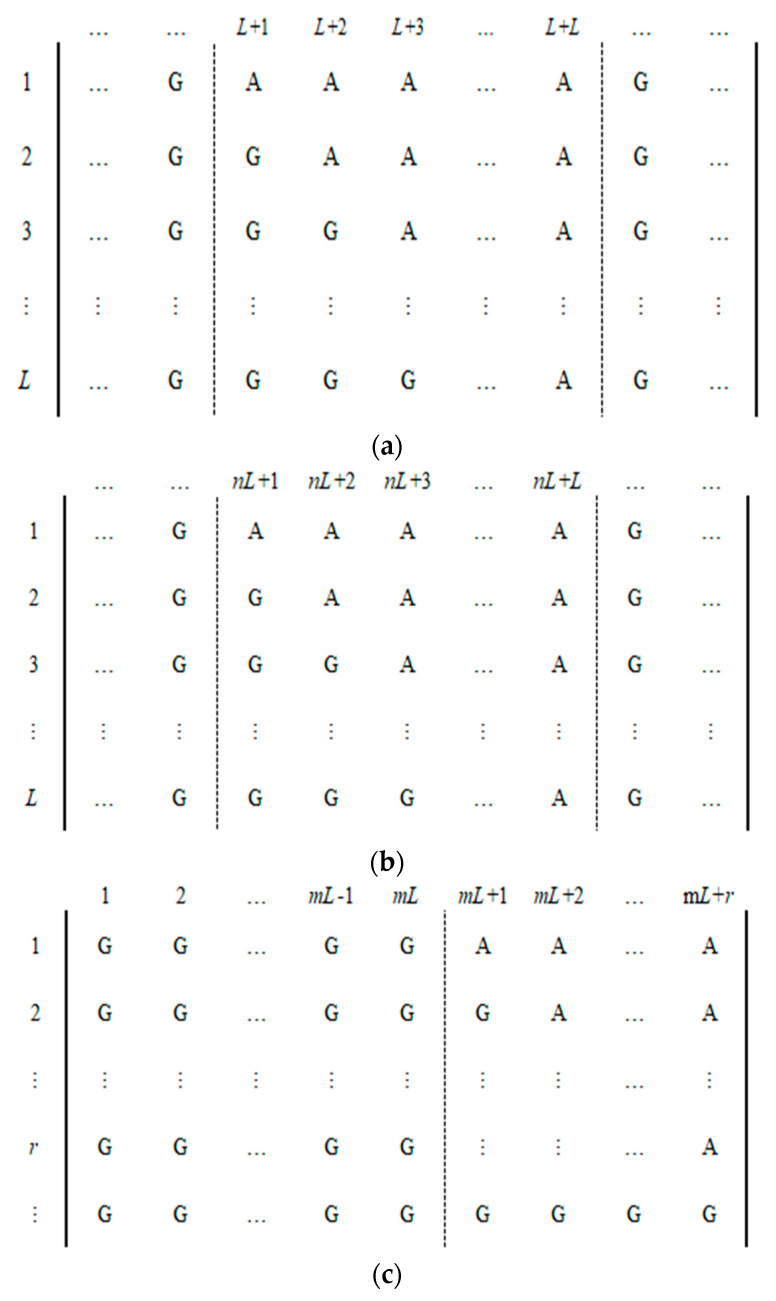
The encoded images of *m* chosen-plaintext images for the case *M* < 4*N*. (**a**) The first encoded image. (**b**) The *n*-th encoded image. (**c**) The last encoded image (0 ≤ *r* < *L*).

**Step 3:** If *L* = *M* < 4*N*, then let *m* = ⌈4×*N*/*L*⌉ − 1, *r* = 4*N* − *m*×*L*, and continue to select *m* plaintext images; each encoded image *P_e_* has the forms as shown in Figure 3.

In Figure 3, each matrix *P_e_* is divided into sub-blocks with continuous *L* columns; the last sub-block may be less than *L* columns. There is only one sub-block in *P_e_*, as each selected plaintext image has both the character “A” and “G”, and the elements of the remaining sub-blocks are all “G”. If *r* > 0, then the number of “A” in the last column of the last chosen-plaintext image is *r*. If *r* = 0, then the number of “A” in the last column of the last chosen-plaintext image is *L*. By using one of the *m* chosen plaintext images, one can obtain *L* or *r* elements of *ly*. The pseudo code of the algorithm in Step 3 is as follows:

*m* ← ⌈4×*N*/*L*⌉−1; *r* = 4*N* − *m*×*L*;

for *n* ← 1: *m*

  *P_e_* ← char(ones(*M*, 4*N*)*‘G’);

  *h* ← *L*;

  if (*r* > 0)&(*n* = *m*)

   *h* ← *r*;

  end if

  for *j* ← *n*×*L*+1:*n*×*L*+*h*

     *P*_e_(1:*j*-*n*×*L*, *j*) ← ‘A’;

  end for *j*

  *P* ← Do DNA decode on *P_e_* with rule 3;

  *C* ← Encrypt *P* by using the encryption machine of original algorithm;

  *P_c_* ← Do DNA encode on *C* with rule 4;

  *P_s_* ← Do DNAxor with *P_c_* and *K_s_*;

  for *j* ← 1:4*N*

    *n_j_* ← The number of “A” in column *j* of *P_s_*;

    if *n_j_* > 0

     *ly*(*j*) ← *n*×*L*+*n_j_*;

    end if

  end for *j*

end for *n*

**Figure 4:**
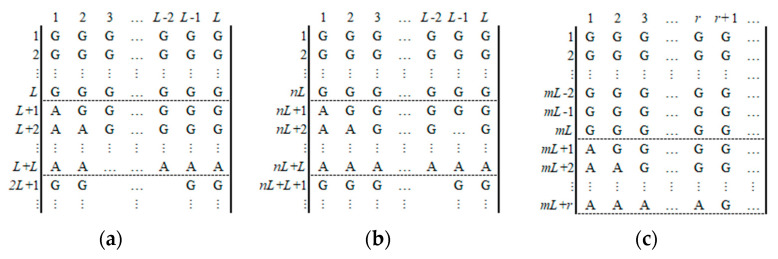
The encoded images of *m* chosen-plaintext images for the case *M* > 4*N*. (**a**) The first encoded image. (**b**) The *n*-th encoded image. (**c**) The last encoded image (0 ≤ *r* < *L*).

**Step 4:** If *L* = 4*N < M*, then let *m* = ⌈*M*/*L*⌉ − 1, *r* = *M* − *m*×*L*, and continue to select *m* plaintext images, whos encoded images have the forms similar to Figure 4.

In Figure 4, each matrix *P_e_* is divided into sub-blocks with continuous *L* rows; the last sub-block may be less than *L* rows. There is only one sub-block in *P_e_* for each selected plaintext image which has both the character “A” and “G”, and the elements of the remaining sub-blocks which are all “G”. If *r* > 0, then the number of “A” in the last row of the last chosen-plaintext image is *r*. If *r* = 0, then the number of “A” in the last row of the last chosen-plaintext image is *L*. By using one of the *m* chosen plaintext images, one can obtain *L* or *r* elements of *lx*. The pseudo code of the algorithm in Step 4 is as follows:

*m* ← ⌈*M*/*L*⌉−1; *r* = *M* − *m* × *L*;

 for *n* ← 1:*m*

  *P_e_* ← char(ones(*M*, 4*N*)*‘G’);

  *h* ← *L*;

  if (*r* > 0)&(*n* = *m*)

   *h* ← *r*;

  end if

  for *i* = *n*×*L*+1:*n*×*L*+*h*

     *P_e_*(*i*, 1:*i*−*n*×*L*) ← ‘A’;

  end for *i*

   *P* ← Do DNA decode on *P_e_* with rule 3;

   *C* ← Encrypt *P* by using the encryption machine of original algorithm;

   *P_c_* ← Do DNA encode on *C* with rule 4;

   *P_s_* ← Do DNAxor with *P_c_* and *K_s_*;

   for *i* ← 1:*M*

     *n_i_* ← The number of “A” in row *i* of *P_s_*;

     if *n_i_* > 0

      *lx*(*i*) ← *n*×*L*+*n_i_*;

     end if

   end for *i*

end for *n*

### 3.3. Decryption the Target Cipher Image CI

Using the decoded key matric *K*_s_, permutation array *lx* and *ly*, the target ciphertext image can be decrypted. The pseudo code of the algorithm to decryption *CI* is as follows:

 *P_c_* ← Do DNA encode on *CI* with rule 4;

  *P_s_* ← Do DNAxor with *P_c_* and *K_s_*;

  for *i* ← 1:*M*

    for *j* ← 1:4*N*

     *P_e_*(*lx*(*i*), *ly*(*j*)) ← *P_s_*(*i*, *j*);

    end for *j*

   end for *i*

  *P* ← Do DNA decode on *P_e_* with rule 3;

According to the algorithm described above, we can see that the number of chosen-plaintext images needed to decipher *lx* and *ly* may be 1, or 1+(⌈4×*N*/*M*⌉−1) = ⌈4×*N*/*M*⌉, or 1 + (⌈*M*/(4×*N*)⌉ − 1) = ⌈*M*/(4×*N*)⌉. Therefore, the total number of chosen-plaintext images needed to decipher all the secret keys of {*K*s, *lx*, *ly*} are 2, or ⌈4×*N*/*M*⌉+1, or ⌈*M*/(4×*N*)⌉+1, respectively.

## 4. Simulation Experiments for Deciphering

To verify the effectiveness of the proposed chosen-plaintext attack algorithm, we give some experimental results on several representative images with different sizes. The secret key parameters of the cryptosystem are set as *x*_0_ =0.3, *y*_0_ = −0.4, *z*_0_ = 1.2, *w*_0_ = 1.0, *a* = 36, *b* = 3, *c* = 28, *d* = 16, and *g* = 0.2.

**Figure 5:**
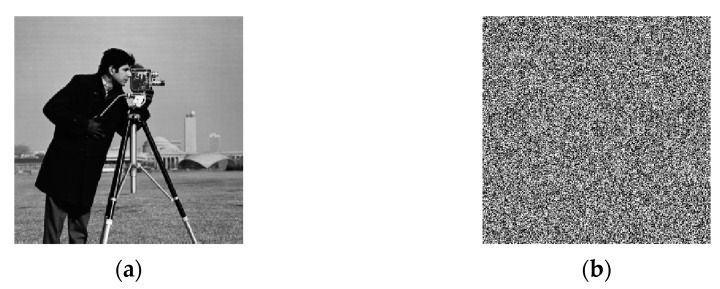
The natural plaintext image and its ciphertext image. (**a**) The plaintext image. (**b**) The ciphertext image of (**a**).

**Figure 6:**
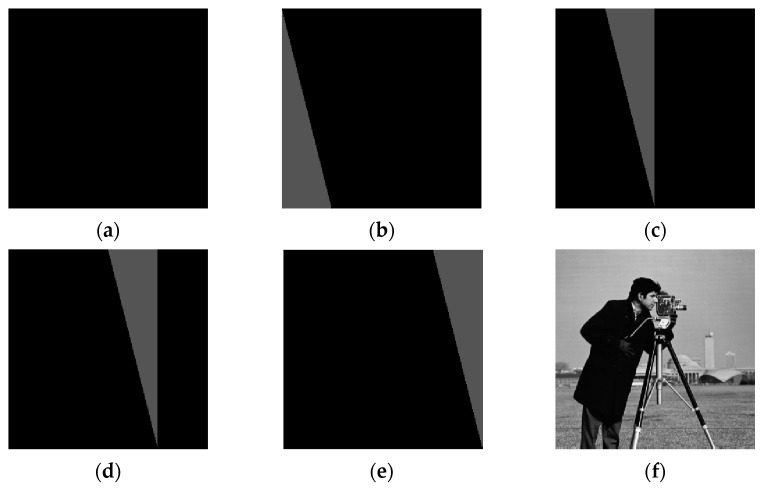
The five chosen-plaintext images and the cracked image. (**a**) The first chosen-plaintext image. (**b**) The second chosen-plaintext image. (**c**) The third chosen-plaintext image. (**d**) The fourth chosen-plaintext image. (**e**)The fifth chosen-plaintext image. (**f**) The cracked image.

**Case 1:** *M* < 4*N*. The original image is a meaningful natural image with a size of 256×256, which is shown in Figure 5a, and its corresponding ciphertext image is shown in Figure 5b. In this case, *M* = 256, *N* = 256, and the total number of chosen-plaintext images needed to decipher the target cipher image of Figure 5b is ⌈4×*N*/*M*⌉+1 = 5. The five chosen-plaintext images are shown from Figure 5e and Figure 6a, respectively. The cracked image is shown in Figure 6f, which coincides with Figure 5a.

**Figure 7:**
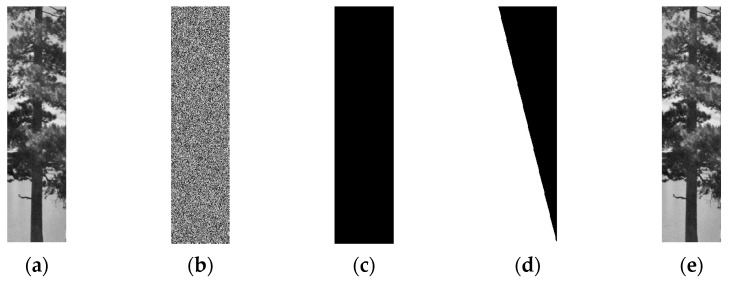
The chosen-plaintext attack on case of *M* = 4*N*. (**a**) The plaintext image. (**b**) The ciphertext image of (**a**). (**c**) The first chosen-plaintext image. (**d**) The second chosen-plaintext image. (**e**) The cracked image.

**Case 2:** *M* = 4*N*. The original image is a meaningful natural image with a size of 512×128, which is shown in Figure 7a, and its corresponding ciphertext image is shown in Figure 7b. In this case, *M* = 512, *N* = 128, and the total number of chosen-plaintext images needed to decipher the target cipher image of Figure 7b is ⌈4×*N*/*M*⌉+1 = 2. The two chosen-plaintext images are shown in Figure 7c,d, respectively. The cracked image is shown in Figure 7e, which coincides with Figure 7a.

**Figure 8:**
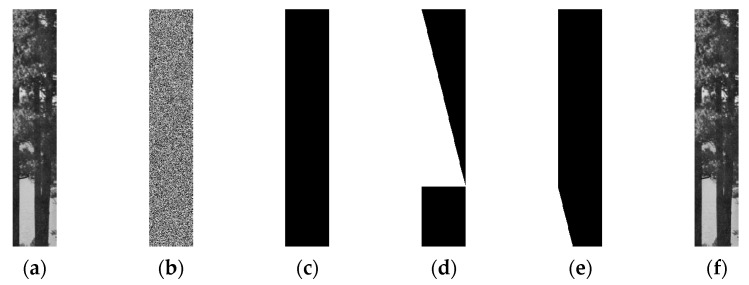
The chosen-plaintext attack on case of *M* > 4*N*. (**a**) The plaintext image. (**b**) The ciphertext image of (**a**). (**c**) The first chosen-plaintext image. (**d**) The second chosen-plaintext image. (**e**) The third chosen-plaintext image. (**f**) The cracked image.

**Case3:** *M* > 4*N*. The original image is a meaningful natural image with a size of 512×96, which is shown in Figure 8a, and its corresponding ciphertext image is shown in Figure 8b. In this case, *M* = 512, *N* = 96, and the total number of chosen-plaintext images needed to decipher the target cipher image of Figure 8b is ⌈*M*/(4×*N)*⌉+1 = 3. The three chosen-plaintext images are shown in Figure 8c–e, respectively. The cracked image is shown in Figure 8f, which coincides with Figure 8a.

**Case4:** A simple numerical example in the case of *M* = 4*N*. The original image is a simple image with a size of 8×2, whose matrix is shown in Equation (7) and the matrix of its corresponding ciphertext image is shown in Equation (8). In this case, *M* = 8, *N* = 2, and the total number of chosen-plaintext images needed to decipher the target cipher image of Equation (8) is ⌈4×*N*/*M*⌉+1 = 2. The two chosen-plaintext images are shown in Equations (9) and (10), respectively. The cracked image is shown in Equation (11), which coincides with Equation (7).

P0 = [11, 12; 21, 22; 31, 32; 41, 42; 51, 52; 61, 62; 71, 72; 81, 82](7)

CI = [162, 180; 20, 112; 126, 41; 193, 245; 199, 255;108, 6; 41, 127; 128, 134](8)

P1 = [0, 0; 0, 0; 0, 0; 0, 0; 0, 0; 0, 0; 0, 0; 0, 0](9)

P2 = [64, 0; 80, 0; 84, 0; 85, 0; 85, 64; 85, 80; 85, 84; 85, 85](10)

P = [11, 12; 21, 22; 31, 32; 41, 42; 51, 52; 61, 62; 71, 72; 81, 82](11)

**Case5:** A simple numerical example in the case of *M* > 4*N*. The original image is a simple image with a size of 9×2, whose matrix is shown in Equation (12) and the matrix of its corresponding cipher image is shown in Equation (13). In this case, *M* = 9, *N* = 2, and the total number of chosen-plaintext images needed to decipher the target ciphertext image of Equation (13) is ⌈*M*/(4×*N*)⌉+1 = 3. The three chosen-plaintext images are shown from Equations (14)–(16), respectively. The cracked image is shown in Equation (17), which coincides with Equation (12).

P0 = [11, 12; 21, 22; 31, 32; 41, 42; 51, 52; 61, 62; 71, 72; 81, 82; 91, 92](12)

CI = [172, 180; 162, 180; 20, 112; 126, 41; 193, 245; 199, 255; 108, 6; 41, 127; 128, 134](13)

P1 = [0, 0; 0, 0; 0, 0; 0, 0; 0, 0; 0, 0; 0, 0; 0, 0; 0, 0](14)

P2 = [64, 0; 80, 0; 84, 0; 85, 0; 85, 64; 85, 80; 85, 84; 85, 85; 0, 0](15)

P3 = [0, 0; 0, 0; 0, 0; 0, 0; 0, 0; 0, 0; 0, 0; 0, 0; 64, 0] (16)

P = [11, 12; 21, 22; 31, 32; 41, 42; 51, 52; 61, 62; 71, 72; 81, 82; 91, 92](17)

## 5. Conclusions

In this paper, the security of a novel image fusion encryption algorithm, based on DNA coding and a hyper-chaotic system, was analyzed in detail. We find that the key stream has nothing to do with the plaintext image and that the plaintext image can be finally cracked by a chosen-plaintext attack. The chosen-plaintext attack algorithm is described with intuitive and clear expression. In our attack algorithm, it only needs ⌈4×*N*/*M*⌉+1 (if 4*N* > *M*) or ⌈*M*/(4×*N*)⌉+1 (if 4*N* < *M*) chosen-plaintext images to crack the target ciphertext image, especially in the case of *M* = 4*N* which only needs two chosen-plaintext images. It is clear that the number of chosen-plaintext images needed to crack the target ciphertext image in our scheme is much less than those used in other attacking algorithms. The effectiveness of the proposed attack algorithm is demonstrated by simulation experiments of typical examples. As a conclusion, the proposed chosen-plaintext attack algorithm is feasible and has a higher attack efficiency.

## Figures and Tables

**Table 1:** Eight DNA encoding rules.

Rules	1	2	3	4	5	6	7	8
A	00	00	01	01	10	10	11	11
T	11	11	10	10	01	01	00	00
G	01	10	00	11	00	11	01	10
C	10	01	11	00	11	00	10	01

**Table 2:** The newly defined XOR operation rules for DNA operation.

XOR	A	G	C	T
A	G	A	T	C
G	A	G	C	T
C	T	C	G	A
T	C	T	A	G

## Data Availability

Data sharing not applicable.
